# The French Ankle Ligament Reconstruction - Return to Sport after Injury (ALR-RSI-Fr) is a valid scale for the French population

**DOI:** 10.1186/s40634-022-00463-1

**Published:** 2022-03-21

**Authors:** Nahi Ajaka, Pierre-Alban Bouché, Michel Dagher, Ronny Lopes, Thomas Bauer, Alexandre Hardy

**Affiliations:** 1 Service de chirurgie orthopédique et traumatologique, Hôpital Ambroise Pare, Paris, France; 2 Service de chirurgie orthopédique et traumatologique, Hôpital Lariboisière, Paris, France; 3 Service de chirurgie orthopédique et traumatologique, Hôpital Henri-Mondor, Paris, France; 4 Service de chirurgie orthopédique et traumatologique, Polyclinique de l’Atlantique, Nantes, France; 5 Clinique du Sport, 36 boulevard Saint-Marcel, 75005 Paris, France

## Abstract

**Purpose:**

The aim of this study was to translate, adapt and validate in French the Ankle Ligament Reconstruction - Return to Sport after Injury (ALR-RSI), a 12-item English language scale assessing the psychological impact of returning to sports after ACL reconstruction. Our hypothesis was that the French version of the ALR-RSI scale would be valid and adaptable to the French population.

**Methods:**

The procedure followed the guidelines for the intercultural adaptation of the self-administered questionnaires. A version of the ALR-RSI-Fr questionnaire was validated according to the international guidelines of COSMIN (COnsensus-based Standards for the selection of health status Measurement INstruments). A prospective study was conducted between March 2020 and June 2021. The study included two population groups, the first one being that of adult athletes of all levels who underwent arthroscopic ankle ligament reconstruction and the second being that of adult athletes who did not suffer from any ankle problems. After consent, patients completed three questionnaires, the ALR-RSI-Fr, the AOFAS and the Karlsson score.

**Results:**

The study included 30 patients and 30 controls who were all athletes. The mean age of the patients was 34.6 with 60% male and 40% female. The average time for patients to return to sport was 5.6 months. Twenty-nine patients (96.6%) underwent arthroscopic ankle reconstruction while only one was eligible for ligament reinsertion. The control group with demographic data matched to the patient group was included in this study.

Correlations between ALRSI, the total Karlsson score and its different sub items and the AOFAS were estimated using Spearman coefficients. Discriminant validity was tested between the “patient” and “control” groups and sub-groups using the Wilcoxon tests. Reliability was evaluated on the ρ intraclass correlation coefficient (ICCC). A strong correlation was noted between the ALR-RSI-Fr , Karlsson and AOFAS with a Spearman score of 0.90 [0.76-0.96].A highly significant difference was found between the "patient" and "control" groups. The internal consistency of the questionnaire was excellent with a Cronbach's alfa of 0.94. Reproducibility was "excellent" with an interclass correlation coefficient of q = 0.97 [0.94-0.99].

**Conclusion:**

This study showed that the cross-cultural adaptation of the English version of the ALR-RSI was successful and validated for the French-speaking population. The discriminant capacity of the scale between patients who underwent reconstruction and healthy subjects was also confirmed. This questionnaire will allow surgeons to better evaluate the psychological impact of returning to sports after ankle ligament reconstruction in French speaking patients

**Level of evidence:**

LEVEL II: Prospective cohort study (patients enrolled at different points in their disease) Control arm of randomized trial.

## Introduction

Ankle sprains remain one of the most frequent causes of emergency room admissions. Representing 6000 cases per day in France [6], ankle sprains are a true economic burden estimated at about $2 billion a year in the United States [16].

The major concern of patients who have benefited from ankle stabilisation is the return to sports at their initial level. However, this is not the case in a significant number of patients despite having satisfactory functional result [8, 10, 13]. Hence the question of the influence of the psychological state of patients on the return to sport at the same initial level in athletes that undergo surgery for lateral ankle stabilisation arises. Questionnaires have been developed to analyse the psychological readiness of operated patients to resume sports following sports injuries. The anterior cruciate ligament reconstruction-return to sports after injury (ACL-RSI), a 12-item questionnaire, was developed to assess the psychological aspect in athletes who benefited from ACL reconstruction before their return to sport. This scale helps measure the athlete’s emotions and confidence in sports performance following surgery [2].

Multiple grading scales of lateral ankle ligament lesions were developed in order to help surgeons in selecting the optimal surgical technique. The arthroscopic classification of chronic ATFL lesions developed by Thes et al. in 2018 that classify ligamentous injuries into five grades ranging from normal ATFL (grade 0) to bald malleolus (grade 4) [19].

The Ankle ligament reconstruction-return to sports after injury (ALR-RSI) is a valid and reproducible scale that helps identify patients that are ready to resume the same sport after ankle ligament reconstruction. This scale can help predict athletes who will find difficulties resuming their respective sport activities [15]. The main goal of this study was the translation, cross-cultural adaptation and validation of the French version of the ALR-RSI. Our hypothesis is that the French version of the ALR-RSI scale is valid and adaptable to the French population. Once valid, this questionnaire will allow surgeons to better evaluate the psychological impact of returning to sports after ankle ligament reconstruction in French speaking patients.

## Methods

Translation and cross-cultural adaptation procedure:

The procedure followed international guidelines for the cross-cultural adaptation of self-administered questionnaires [1]. After receiving the author’s agreement, which had been approved by the institutional review board in advance (IRB:COS-RGDS-2021-07-003-HARDY-A) the questionnaire was translated from English to French by two orthopaedic surgeons that were both native French speakers. A first French draft of the questionnaire was chosen following a consensus meeting. This draft was then translated back to English by two native English speakers who were not aware of the original version. A pilot version of the questionnaire was then formulated following a final consensus meeting. The latter was then administered to 10 randomly chosen athletes who benefited from an arthroscopic ankle ligament reconstruction. A final version of the questionnaire was then formulated taking into consideration the sample population’s remarks.

The study included 30 patients and 30 controls. The average age of the study population, that included 60% of men and 40% of women, was 34.6. All patients were athletes of which 13.8% were professional (Table 1). The average time patients needed before returning to sports activity was 5.6 months. Twenty-nine patients (96.6%) benefited from arthroscopic ankle ligament reconstruction while only one patient was eligible for arthroscopic ligament reinsertion following the classification of Thes et al. of 2018 [19] (Table 1). The control group had demographic data that matches the patient group. (Table 2).

**Table 1:** Demographic presentation of the patient population

Parameters	Values	N	statistics
		30	
Age (years)		30	34.6 (10.2)
Gender	Female	12	40%
	Males	18	60%
Side	Right	16	53.3%
	Left	14	46.7%
Sport	Box	1	3.4%
	Running	15	51.7%
	Cycling	1	3.4%
	Horse-back riding	1	3.4%
	Climbing	2	6.8%
	Football	4	13.7%
	Judo	1	3.4%
	Tennis	4	13.8%
Sport Level	Amateur	25	86.2%
	Professionel	4	13.8%
Surgical procedure	Reconstruction	28	96.6%
	Reinsertion	1	3.4%
Time before returning to sports (months)		29	5.6 (2.5)
Return to sports at the same level	Non	9	32.1%
	Oui	19	67.9%
ALR-RSI total		30	83.1 (19.7)
AOFAS total		30	78.9 (11.9)
Karlsson total		30	70.8 (12.5)

**Table 2:** Demographic Comparison of the two study populations

Parameters	Values	N	Statistique*	N	Statistique*	*p*-value
		30	Control	30	Intervention	
Age (years)		30	32.8 (9.711)	30	34.63 (10.19)	0.40
Gender	F	15	50%	12	40%	0.60
	M	15	50%	18	60%	
Side	Right	15	50%	16	53.3%	1.00
	Left	15	50%	14	46.7%	
Sport	Gymnastics	3	10%	0	0%	0.065
	Dancing	1	3.3%	0	0%	
	Basketball	2	6.7%	0	0%	
	Boxing	1	3.3%	1	3.4%	
	Running	20	66.7%	15	51.7%	
	Cycling	0	0%	1	3.4%	
	Horse-back riding	0	0%	1	3.4%	
	Climbing	1	3.3%	2	6.8%	
	Football	0	0%	4	13.7%	
	Judo	0	0%	1	3.4%	
	Tennis	1	3.3%	4	13.8%	
	Yoga	1	3.3%	0	0%	
Sport level	Amateur	27	90%	25	86.2%	0.71
	Professionel	3	10%	4	13.8%	
ALR-RSI total		30	110.7 (17.34)	30	83.13 (19.73)	**< 0.0001**
AOFAS total		30	95.93 (8.111)	30	78.9 (11.94)	**< 0.0001**
Karlsson test total		30	86.8 (6.217)	30	70.8 (12.48)	**< 0.0001**

### Validity and reliability of the final ACL-RSI-Fr

The final version was validated according to COSMIN international guidelines (COnsensus based Standards for the selection of health status Measurement INstruments) [11]. A prospective study was performed from June 2020 till June 2021.

The study population included two groups, the first being adult athletes of all levels who benefited for an arthroscopic ankle ligament reconstruction or reinsertion depending on the grade of the ATFL lesion according to the classification of Thes et al. [19] and the second one being composed of adult athletes with no history of ankle instability. Non-athletes and patients with other concomitant injuries were excluded from this study (OCD, Valgus of the hindfoot). The reference scales that were used are: The American Orthopedic Foot & Ankle Society (AOFAS) [9] and the Karlsson score [12].

After obtaining their consent all patients were contacted. They were asked to fill out the three questionnaires: the ALR-RSI-Fr the AOFAS and the Karlsson score. The ALR-RSI-Fr score was filled twice at 3 to 4 days interval by the operated group and only once by the control group.

### Statistical analysis

To describe quantitative variables, the mean and standard deviation (SD) were used. To describe dichotomous variables, their number of events and their percentage were used. A sample size of 30 produces a two-sided 95% confidence interval with a width smaller than 0.38 when the estimate of Spearman’s rank correlation is above 0.75. To estimate the correlations between ALRSI, the total Karlsson score and the AOFAS, Spearman coefficients were used. If the coefficient was *r* > 0.5 the correlation was considered «strong», “moderate” if 0.5 < *r* < 0.3 or “weak” if 0.3 < *r* < 0.1. A Wilcoxon test was used to compare the “patient” and “control” groups to assess the discriminant validity. We also compared the patients who had returned to their sport level and those who returned at an inferior sport level. The Cronbach alpha coefficient was calculated to estimate the internal consistency and was “excellent” if α ≥ 0.90. The ρ intraclass correlation coefficient (ICCC) was used to evaluate the reliability. The reproducibility was “excellent” (*ρ* > 0.75) or “good” (0.75 < *ρ* < 0.40). The percentage of missing responses, the ceiling and floor effects were used to evaluate the feasibility [17]. The statistical analyses were calculated using the R software (version 3.5.0). According to Terwee et al. [17], in the presence of a ceiling or floor effect of more than 15% there is an inherent problem with the validity of the contents when generating questionnaire items.*p* < 0.05 was considered to be significant. All tests were 2-sided. The R software (version 3.5.0) was used to perform the statistical analyses.

## Results

### Cross-cultural adaptation

The French translation and the English back translation did not create any major linguistic problems. Some changes were made to the initial questionnaire after the comments made by the pilot population. The final questionnaire kept an 11-point Likert score in the form of checkboxes from 0 to 10 with 12 questions in total.

### Return to sports

Ninety-six percent of patients returned to sports at an average time of 5.6 months. Of these, only 67.9% returned to sport at the same level.

### Construct validity

The ALR-RSI-Fr questionnaire appears to have a significantly positive correlation with the reference scales used. A strong correlation was noted between the ALR-RSI-Fr, Karlsson and AOFAS with a Spearman score of 0.90 (0.76–0.96). (Tables 3 and 4).

**Table 3:** Correlation between ALR-RSI & Karlsson

Coefficient	ALR-RSI (/100)	Karlsson total (/100)	Pain	Swelling	Instability	Stiffness	Stairs climbing	Running	Working activity	Support
	83.1 (19.7)	70.8 (12.5)	15.5 (3.6)	7.5 (3.4)	11.7 (3.3)	3.9 (2.7)	9.7 (1.3)	7.2 (2.5)	11 (2.4)	4 (1.4)
Spearman		0.85 [0.66–0.94]	0.74 [0.49–0.87]	0.52 [0.17–0.75]	0.64 [0.30–0.82]	0.24 [−0.16–0.55]	0.33 [0.16–0.70]	0.47 [0.04–0.70]	0.55 [0.30–0.74]	0.44 [0.06–0.70]

**Table 4:** Correlation between ALR-RSI and AOFAS

Coefficient	ALR-RSI (/100)	AOFAS tot (/100)
	83.1 (19.7)	78.9 (11.9)
Spearman		0.90 [0.76–0.96]

### Discriminant validity

A highly significant difference was found between the “patient” and “control” groups. Also, a significant difference was found between the subgroups of patients that resumed the sport at the same level as compared to those who returned to sport at a lower level with an ALR-RSI-Fr score of 91.6(14.9) and 72.7(15.9) respectively (*P* value< 0.01).

### Internal consistency

The internal consistency of the questionnaire, based on the strength of correlation between its 12 items, was excellent with a Cronbach’s alfa of 0.94.

### Reliability

**Fig. 1:**
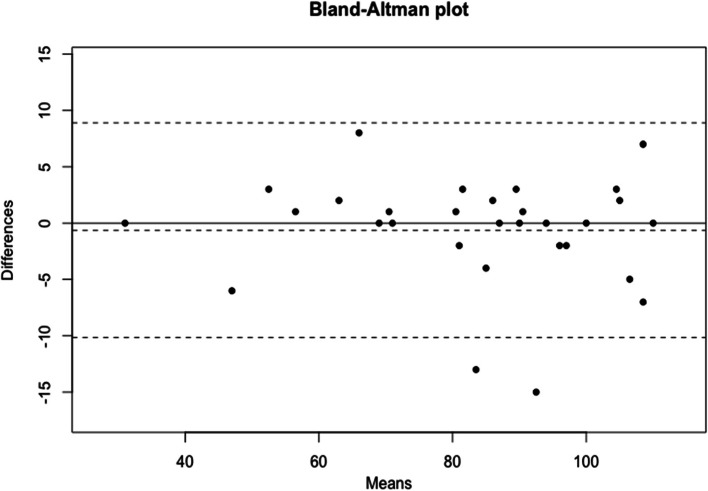
Reproducibility (test–retest) of the ALR-RSI-Fr: Bland and Altman diagram showing good agreement between the two measurements and no systematic bias

The reproducibility of the questionnaire was analyzed by a test-retest (Fig. 1). The average ALR-RSI-Fr score when it was first filled was 83.1 ± 19.7, while it was 83.8 ± 20.2 the second time. The correlation between the two versions was “strong” with a Pearson correlation coefficient of 0.90. Reproducibility was “excellent” with an interclass correlation coefficient of q = 0.97 [0.94–0.99].

### Feasibility

Patients responded to all questions, no missing answers were reported. The average questionnaire completion time was 2.3 min. For the floor effect corresponding to the percentage of patients that answered with a score of 0 for each question ranged from 0% to 3.3%. Meanwhile the ceiling effect, corresponding to the percentage of patients that answered with a score of 10 for each question, ranged from 3.3% to 9.7%.

## Discussion

The most relevant conclusion that could be drawn from this study is that the French version of the ALR-RSI questionnaire is valid, reproducible and comparable to the English version.

Valid and reliable rating scales are used to assess the severity of a functional problem in patients. They provide a common language for surgeons and researchers and facilitate the comparison of their results for different treatment modalities.

The ideal scale would be one that proved its reliability and validity so that it can be applied in everyday life. An accurate and reliable scale helps in detecting significant changes that patients feel. They may include subjective (patient perception of pain and function) or objective (physical or radiographic examination) data, or both.

The ALR-RSI-Fr questionnaire was compared to two questionnaires: the AOFAS and the Karlsson score. The AOFAS score was chosen because it remains the most commonly used questionnaire by French orthopaedic surgeons for the evaluation of patients suffering from ankle or hind-foot injuries. This questionnaire evaluates both ankle function and pain, which are two critical points for patients operated of an ankle ligament reconstruction who wish to resume their sport at the same level as before [14, 3].

The Karlsson Ankle Functional Score (KAFS) has satisfactory sensitivity in assessing the functional abilities of patients after an ankle sprain [18]. The Karlsson scale allows the assessment of symptoms, ambulation, activity level and the use of a splint during physical activity in a single aggregate score.

The ALR-RSI scale is the first score that specifically assesses the psychological impact on returning to sports after an ankle ligament reconstruction. The discriminating value of the ACL-RSI-Fr was also confirmed. This questionnaire correctly distinguished patients who underwent ankle ligament reconstruction from patients without any injury to the ankle. The ALR-RSI-Fr score was significantly higher in the control group and for patients who have resumed their sport at the same initial level. This scale has therefore made it possible to successfully assess the return to sport in the target population.

The time it takes before an athlete can return to sports after an ankle ligament reconstruction may vary from one patient to another. This questionnaire may allow the surgeon to assess the patient’s ability to resume his sports activity [5, 7].

The ALR-RSI is a valid and reliable questionnaire, which makes possible the identification of patients who are psychologically ready to resume their sport, after an ankle ligament reconstruction of the ankle [12].

Results obtained in this study are comparable to the ones found in the literature. Similarly, Bohu et al. found a strong correlation between the ACL-RSI-Fr all the reference scales used [2].

The importance of this subject has made it necessary to translate, adapt and validate a French version of the ALR-RSI scale.

A positive and significant correlation was found between the ALR-RSI-Fr and the other two questionnaires used.

In our study 96% of patients returned to sports at an average time of 5.6 months. Of these, only 67.9% returned to sport at the same level. The fact that the percentage of patients that returned to sports after surgery is slightly higher than the literature might be due to the small sample size [4]. The essential limitation of this study is the short duration of follow-up and the limited number of patients. A study with a longer follow-up and bigger sample size would lead to a better analysis of the evolution of patients who have not resumed sport at the same level yet.

## Conclusion

The French version of the ALR-RSI scale was found to be valid, consistent, reproducible and comparable to the English version and can therefore be used by orthopaedic surgeons in France. This score will make possible the assessment of the psychological impact regarding the return to sport of French patients who undergo surgery for chronic ankle instability. Nonetheless, a study with a larger sample size and longer follow-up could help make a stronger scientific conclusion.

## References

[1] Beaton D, Bombardier C, Guillemin F, Ferraz M (2000). Guidelines for the process of cross-cultural adaptation of self-report measures. Spine.

[2] Bohu Y, Klouche S, Lefevre N, Webster K, Herman S (2014). Translation, cross-cultural adaptation and validation of the French version of the Anterior Cruciate Ligament-Return to Sport after Injury (ACL-RSI) scale. Knee Surg Sports Traumatol Arthrosc.

[3] Button G, Pinney S (2004). A meta-analysis of outcome rating scales in foot and ankle surgery: is there a valid, reliable, and responsive system?. Foot Ankle Int.

[4] Cordier G, Ovigue J, Dalmau-Pastor M, Michels F (2020). Endoscopic anatomic ligament reconstruction is a reliable option to treat chronic lateral ankle instability. Knee Surg Sports Traumatol Arthrosc.

[5] Guillo S, Odagiri H, van Rooij F, Bauer T, Hardy A (2021). All-inside endoscopic anatomic reconstruction leads to satisfactory functional outcomes in patients with chronic ankle instability. Knee Surg Sports Traumatol Arthrosc.

[6] Hølmer P, Søndergaard L, Konradsen L, Nielsen P, Jørgensen L (1994). Epidemiology of Sprains in the Lateral Ankle and Foot. Foot Ankle International.

[7] Li H, Hua Y, Li H, Chen S (2020). Anatomical reconstruction produced similarly favorable outcomes as repair procedures for the treatment of chronic lateral ankle instability at long-term follow-up. Knee Surg Sports Traumatol Arthrosc.

[8] Li H, Hua Y, Li H, Ma K, Li S, Chen S (2017). Activity Level and Function 2 Years After Anterior Talofibular Ligament Repair: A Comparison Between Arthroscopic Repair and Open Repair Procedures. Am J Sports Med.

[9] Kitaoka H, Alexander I, Adelaar R, Nunley J, Myerson M, Sanders M (1994). Clinical rating systems for the ankle-hindfoot, midfoot, hallux, and lesser toes. Foot Ankle Int.

[10] Lopes R, Andrieu M, Cordier G, Molinier F, Benoist J, Colin F, Thès A, Elkaïm M, Boniface O, Guillo S, Bauer T (2018) French Arthroscopic Society. Arthroscopic treatment of chronic ankle instability: Prospective study of outcomes in 286 patients. Orthop Traumatol Surg Res 2018;104(8S):S199–S205. 10.1016/j.otsr.2018.09.005 10.1016/j.otsr.2018.09.005 30245066

[11] Mokkink L, Terwee C, Patrick D, Alonso J, Stratford P, Knol D (2010). The COSMIN study reached international consensus on taxonomy, terminology, and definitions of measurement properties for health-related patient-reported outcomes. J Clin Epidemiol.

[12] Roos E, Brandsson S, Karlsson J (2001). Validation of the foot and ankle outcome score for ankle ligament reconstruction. Foot Ankle Int.

[13] Shawen SB, Dworak T, Anderson RB (2016). Return to play fol- lowing ankle sprain and lateral ligament reconstruction. Clin Sports Med.

[14] Shazadeh Safavi P, Janney C, Jupiter D, Kunzler D, Bui R, Panchbhavi V (2018). A systematic review of the outcome evaluation tools for the foot and ankle. Foot Ankle Specialist..

[15] Sigonney, F., Lopes, R., Bouché, PA. et al*.* (2020) The ankle ligament reconstruction-return to sport after injury (ALR-RSI) is a valid and reproducible scale to quantify psychological readiness before returning to sport after ankle ligament reconstruction. Knee Surg Sports Traumatol Arthrosc 28**,** 4003–4010. 10.1007/s00167-020-06020-6 PMC7669765 32356045

[16] Soboroff S, Pappius E, Komaroff A (1984). Benefits, risks, and costs of alternative approaches to the evaluation and treatment of severe ankle sprain. Clin Orthop Related Res.

[17] Terwee CB, Bot SD, de Boer MR (2007). Quality criteria were proposed for measurement properties of health status questionnaires. J Clin Epidemiol.

[18] Theunissen M, Peters ML, Schouten EG, Fiddelers AA, Willemsen MG, Pinto PR, Gramke HF, Marcus MA (2014). Validation of the surgical fear questionnaire in adult patients waiting for elective surgery. PLoS One.

[19] Thès A, Odagiri H, Elkaïm M, Lopes R, Andrieu M, Cordier G, Molinier F, Benoist J, Colin F, Boniface O, Guillo S, Bauer T, French Arthroscopic Society (2018). Arthroscopic classification of chronic anterior talo-fibular ligament lesions in chronic ankle instability. Orthop Traumatol Surg Res.

